# Marker Assisted Development and Characterization of Herbicide Tolerant Near Isogenic Lines of a Mega Basmati Rice Variety, “Pusa Basmati 1121”

**DOI:** 10.1186/s12284-020-00423-2

**Published:** 2020-09-15

**Authors:** Nitasha Grover, Aruna Kumar, Ashutosh Kumar Yadav, S. Gopala Krishnan, Ranjith Kumar Ellur, Prolay Kumar Bhowmick, K. K. Vinod, Haritha Bollinedi, M. Nagarajan, C. Viswanathan, Amitha Mithra V. Sevanthi, Nagendra Kumar Singh, Trilochan Mohapatra, Ashok Kumar Singh

**Affiliations:** 1 Division of Genetics, ICAR-Indian Agricultural Research Institute, New Delhi, 110012 India; 2 Amity Institute of Biotechnology, Amity University, Noida, Uttar Pradesh 201303 India; 3 Rice Breeding and Genetics Research Centre, ICAR-Indian Agricultural Research Institute, Aduthurai, Tamil Nadu 612101 India; 4 Division of Plant Physiology, ICAR-Indian Agricultural Research Institute, New Delhi, 110012 India; 5 ICAR-National Institute for Plant Biotechnology, New Delhi, 110012 India; 6 Indian Council of Agricultural Research, Krishi Bhawan, New Delhi, 110001 India

**Keywords:** DSR, Marker assisted backcross breeding, *AHAS*, Herbicide tolerant, Foreground and background selection, Grain and cooking quality, Basmati rice

## Abstract

**Background:**

Direct-seeded rice (DSR) is a potential technology for sustainable rice farming as it saves water and labor. However, higher incidence of weed under DSR limits productivity. Therefore, there is a need to develop herbicide tolerant (HT) rice varieties.

**Results:**

We used marker assisted backcross breeding (MABB) to transfer a mutant allele of Acetohydroxy acid synthase (*AHAS*) gene, which confers tolerance to imidazolinone group of herbicides from the donor parent (DP) “Robin” into the genetic background of an elite popular Basmati rice variety, Pusa Basmati 1121 (PB 1121). Foreground selection was done using the *AHAS* gene linked Simple Sequence Repeat (SSR) marker RM6844 and background selection was performed using 112 genome-wide SSR markers polymorphic between PB 1121 and Robin. Phenotypic selection for agronomic, Basmati grain and cooking quality traits in each generation was carried out to improve the recovery of recurrent parent phenome (RPP). Finally, a set of 12 BC_4_F_4_ near isogenic lines (NILs), with recurrent parent genome (RPG) recovery ranging from 98.66 to 99.55% were developed and evaluated. PB 1121-HT NILs namely 1979-14-7-33-99-10, 1979-14-7-33-99-15 and 1979-14-7-33-99-66 were found superior to PB 1121 in yield with comparable grain and cooking quality traits and herbicide tolerance similar to Robin.

**Conclusion:**

Overall, the present study reports successful development of HT NILs in the genetic background of popular Basmati rice variety, PB 1121 by introgression of mutated *AHAS* allele. This is the first report on the development of HT Basmati rice. Superior NILs are being evaluated in the national Basmati trials, the release of which is likely to provide a viable option for the adoption of DSR technology in Basmati rice cultivation.

## Background

In South-East Asian countries, where rice is primarily cultivated by transplanting, DSR holds great promise as it is a potential water and labor saving technology, which offers the possibility of saving huge amount of water and labour needed for puddling and transplanting. However, DSR cultivation suffers from high weed infestation. Weeds pose a serious threat by competing with the crop for water, nutrients and light, thereby hampering productivity (Powles and Yu 2010). In India, upto 30% of the total cost of rice cultivation goes in controlling weeds (Rao et al. 2015). It has been demonstrated that with effective weed management, the yields from DSR is widely comparable to transplanted rice (Pathak et al. 2011). The use of herbicides is the most effective and economical option to control weeds (Anderson and Georgeson 1989). However, the herbicides that are safe and effective at minimal doses should be used to ensure environmental safety. Imidazolinone group of herbicides (imazapyr, imazapic, imazethapyr, imazamox, imazamethabenz and imazaquin etc.), control weeds by inhibiting the enzyme acetohydroxyacid synthase (AHAS), also called acetolactate synthase (ALS). AHAS is a critical enzyme for the biosynthesis of branched-chain amino acids namely, leucine, isoleucine and valine in plants. These herbicides, also have low mammalian toxicity due to lack of *AHAS* gene (Tan et al. 2006; Piao et al. 2018). Due to the above benefits, the herbicides of imidazolinone group are most widely used for weed control in crops like soybean, groundnut etc., which possess natural tolerance to these herbicides. However, the crops like rice, maize, wheat, oilseed rape and sunflower etc. are highly sensitive to imidazolinones. Several variants of *AHAS* genes conferring imidazolinone tolerance have been developed through mutagenesis and selection in different crops and commercialized as Clearfield® crops since 1992. Imidazolinone herbicides control a broad spectrum of grass and broadleaf weeds in imidazolinone-tolerant crops, including weeds that are closely related to the crop itself and some key parasitic weeds (Tan et al. 2005).

Here, we report the transfer of mutant allele of *AHAS* gene conferring herbicide tolerance from Robin into a mega Basmati rice variety, PB 1121, through MABB and assessment of PB 1121 NILs for agronomic, grain and cooking quality performance in addition to herbicide tolerance.

## Material and Methods

### Plant Materials

In this study, we used the herbicide tolerant rice mutant Robin as donor and a mega-Basmati rice variety, PB 1121 as the recurrent parent (RP) for the transfer of HT trait. The mutant Robin was developed from an upland rice variety Nagina 22 (N22) through induced mutagenesis using ethyl methane sulfonate (EMS) (Shoba et al. 2017). PB 1121, developed by our group at ICAR-Indian Agricultural Research Institute (IARI), New Delhi, is a Basmati rice variety par excellence in grain and cooking quality with an exceptionally high cooked kernel length (22 mm) and elongation ratio of > 2.5. This variety is currently grown on approximately 1.2 million ha area (60% of the Basmati area in India) and contributes annually 3.41 billion USD to foreign exchange earnings through the export of Basmati rice (Singh et al. 2018b).

### MABB Strategy for Development of HT Lines

**Fig. 1:**
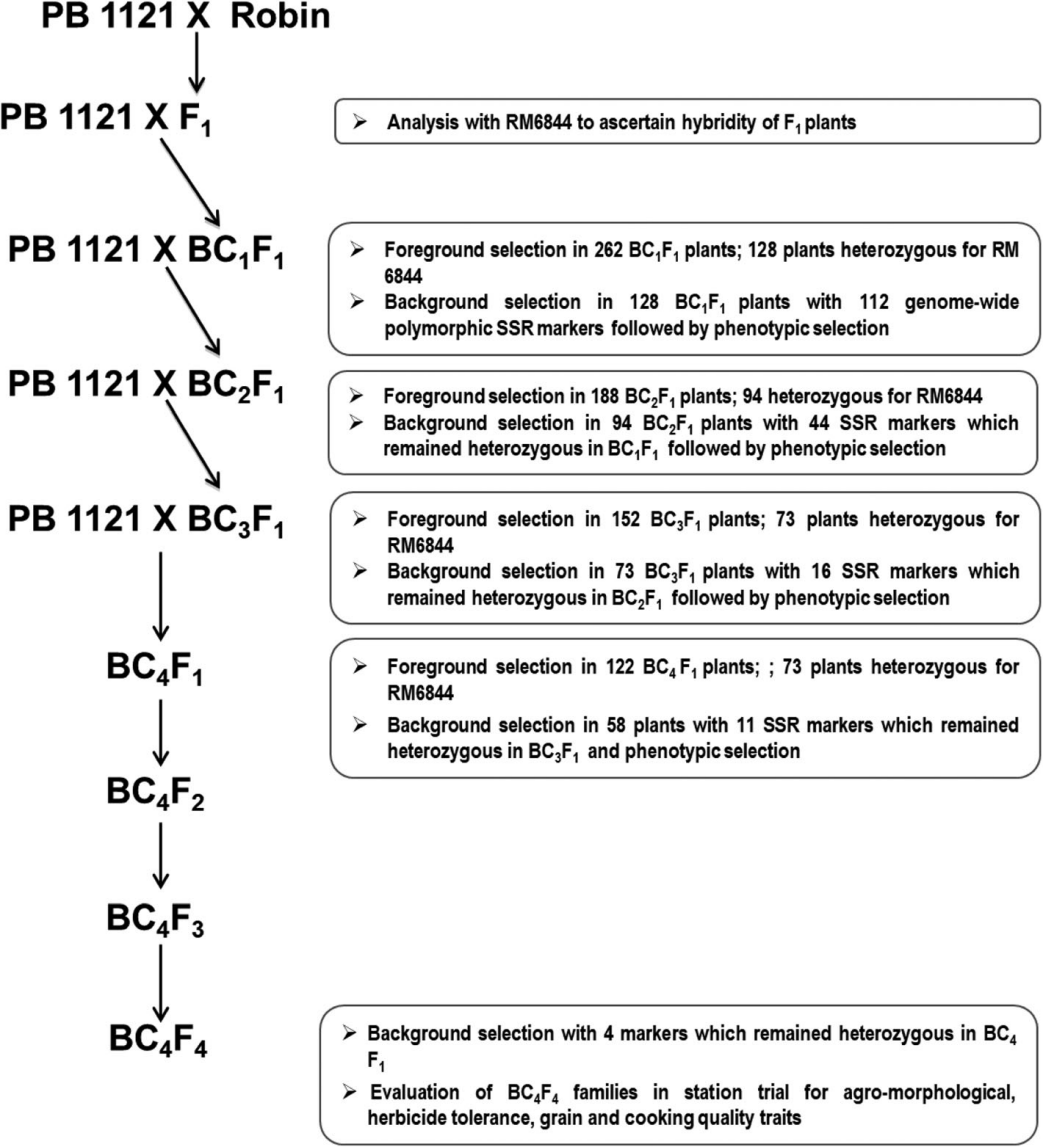
Marker aided backcross breeding strategy used for transferring the herbicide tolerance trait into PB 1121

PB 1121 and mutant Robin were first evaluated for their tolerance to the herbicide “Imazethapyr” at the dose of 2.5 ml/litre. The F_1_ seeds were produced by crossing PB 1121 as female and Robin as male and the hybridity of the F_1_ plants was tested using the SSR marker RM6844 linked with *AHAS* gene. The F_1_s were designated as Pusa 1979 and single F_1_ was backcrossed with PB 1121 to generate BC_1_F_1_ seeds. One BC_1_F_1_ plant with the highest recovery of RPG and recurrent parent phenome (RPP) was backcrossed to PB 1121 to generate the BC_2_F_1_ seeds. A similar strategy was employed till BC_4_F_1_ generation, wherein in each of the generations, the plant heterozygous for the mutant *AHAS* allele along with maximum recovery for RPG and RPP were identified. The superior BC_4_F_1_ plants were advanced to BC_4_F_2_ generation and plants homozygous for the mutant *AHAS* allele were identified. Further, the selected BC_4_F_2_ plants were advanced to BC_4_F_4_ generation via pedigree-based phenotypic selection (Fig.1).

### Molecular Analysis

Total genomic DNA from leaf tissues was extracted using Cetyl Trimethyl Ammonium Bromide (CTAB) method (Doyle 1991). The PCR reaction of a total 10 μl volume was set up which included, 25–30 ng of template DNA, 5 pmol each of the forward and reverse primers and 2X red dye PCR master mix (Genei Laboratories Pvt. Ltd., Bangalore). The program of PCR amplification consisted of initial denaturation at 95 °C for 5 min; 35 cycles of denaturation at 95 °C for 40s, annealing at 58 °C for 40s, extension at 72 °C for1 min; and a final extension at 72 °C for 10 min. The amplified products were resolved on 3.5% Metaphor™ Agarose gel mixed with 0.1 mg/ml ethidium bromide. The amplicons were visualized on ultraviolet trans-illuminator (Gel Doc™ + Imager, Bio- Rad Laboratories Inc., U.S.A).

### Foreground and Background Selection

Foreground selection for the identification of plants carrying the mutant *AHAS* allele was carried out using SSR marker RM6844 linked with *AHAS* gene at a distance of 1.2 cM in chromosome 2 (Shoba et al. 2017), primer details are given in Table 1. For background selection, the primer sequence of genome-wide SSR markers was fetched from the rice marker database of Gramene (http://www.gramene.org). A total of 856 SSR markers were used to identify 112 polymorphic SSR markers between the parents PB 1121 and Robin, for use in background selection (Table 2). Agarose gel image and graphical presentation of all 112 polymorphic SSR markers is given in additional file 1: Figure S1 and additional file 2: Figure S2, respectively. Primers details are provided in additional file 5: Table S1. During the background selection in backcross generations, the homozygous and heterozygous plants for PB 1121 allele at each marker loci were counted separately. A reductionist strategy was considered for this, markers that were found to be homozygous for PB 1121 allele in a given generation were not included in subsequent generations for background selection. RPG recovery was estimated using the formula:

$$ \boldsymbol{RPG}\ \boldsymbol{recovery}\ \left(\%\right)=\frac{\boldsymbol{Number}\ \boldsymbol{of}\ \boldsymbol{markers}\ \boldsymbol{homozygous}\ \boldsymbol{for}\ \boldsymbol{RP}\ \boldsymbol{alleles}+\left(\mathbf{0.5}\ast \boldsymbol{Number}\ \boldsymbol{of}\ \boldsymbol{markers}\ \boldsymbol{heterozygous}\right)}{\boldsymbol{Total}\ \boldsymbol{number}\ \boldsymbol{of}\ \boldsymbol{polymoprhic}\ \boldsymbol{markers}\ \boldsymbol{used}\ \boldsymbol{for}\ \boldsymbol{background}\ \boldsymbol{selection}}\boldsymbol{X}\mathbf{100} $$

**Table 1:** Details of SSR marker linked to the *AHAS* gene which was used in foreground selection

Marker	Primer Sequences	Tm	PB 1121 Allele	Robin Allele	Position
RM6844	F: AGTCCAAGAAAGGCACGAGAGG	58 °C	140 bp	200 bp	1.2 cM
	R: CTGCATCGAAGAAGAAGAAGAAGC

**Table 2:** Details of SSR markers used for parental polymorphic survey

Markers used for	Total No. of Markers surveyed	Total No. of Polymorphic markers	Polymorphic Markers used	Polymorphism (%)
**Foreground**	1	1	1	–
**Background**^**a**^	856	112	112	13.08
**Chromosome 2**	96	18	18	18.75

^a^Includes markers on Chromosome 2

The RPG recovery was visualized using Graphical GenoTypes (GGT) Version 2.0 software (Van Berloo 1999). Based on molecular marker analysis, similarity of NILs to PB 1121 was computed using Jaccard’s coefficient of similarity for generating a dendrogram following an unweighted pair group method with arithmetic mean (UPGMA). Further, for cluster analysis, NTSYS-PC-2.02f (Rohlf 1998) was used.

### Molecular Screening for Aroma Gene

The NILs and the parents were also screened for the presence of *badh2* gene, responsible for aroma in Basmati rice, using gene based marker *‘*nksbad2’ (Amarawathi et al. 2008). Primer details are given in Table 3.

**Table 3:** Details of nks_bad2 gene based SSR marker for aroma

Marker	Primer Sequences	Tm	PB1121 Allele	Robin Allele	Position (Mb)
nks_bad2	F: GGTTGCATTTACTGGGAGTTATG	58 °C	82 bp	90 bp	Gene based
	R: TCCACAGAAATTTGGAAACAAA				

### Screening for Imazethapyr Tolerance

Twelve HT-NILs of PB 1121 along with PB 1121 and Robin were planted in a randomized complete block design (RCBD) with three replications and sprayed with herbicide, Imazethapyr (commercially available as Pursuit™) @ concentration of 2.5 ml/liter, after 10 days of transplanting. Another set of same experimental material was grown side by side and used as unsprayed control with manual weeding. Visual observation of herbicide tolerance of the HT-NILs was made on 15 days after spray as per the standard protocol in rice (Shoba et al. 2017).

### Evaluation of Agro-Morphological, Grain and Cooking Quality Parameters

Agro-morphological evaluation of the HT-NILs and parents was done in RCBD with three replications following recommended agronomic practices. Data on the agro-morphological traits viz*,* days to 50% flowering (DFF), plant height (PH), number of productive tillers per plant (NPT), panicle length (PL), spikelet fertility percent (SF %), thousand grain weight (TGW) were recorded on five plants taken at random from the two middle rows of each plot under both sprayed and unsprayed conditions. The plot yield was recorded in kilogram/hectare (kg/ha) from each replication. The data on grain and cooking quality traits such as hulling percentage (HUL%), milling percentage (MIL%), head rice recovery percentage (HRR%), kernel length before cooking (KLBC), kernel breadth before cooking (KBBC), kernel length after cooking (KLAC), kernel breadth after cooking (KBAC), kernel elongation ratio (ER), alkali spreading value (ASV) (Little 1958) and aroma (Sood and Siddiq 1978) was recorded.

The statistical analysis of agro-morphological data was carried out using CropStat 7.2 (IRRI, *CropStat 7.2* 2014). *Student’s t-test* was performed for statistical significance differences for yield between unsprayed and herbicide sprayed condition at *P* ≤ 0.05.

## Results

### Development of PB 1121-NILs with Herbicide Tolerance

**Fig. 2:**
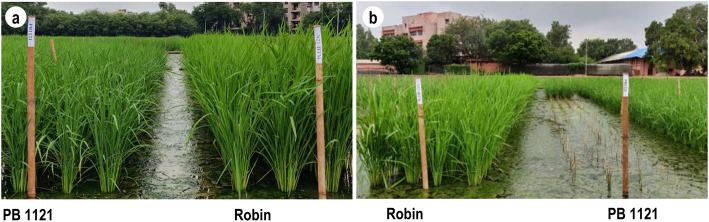
Phenotypic evaluation of donor parent, “Robin” and recurrent parent, “PB 1121” for Imazethapyr herbicide tolerance @ concentration of 2.5 ml/liter, **a** Unsprayed, **b** Sprayed

The recurrent parent PB 1121 and donor Robin were validated for their tolerance to herbicide, Imazethapyr. PB 1121 was highly susceptible and exhibited complete mortality within 15–20 days of herbicide spray, while the donor Robin was highly tolerant to Imazethapyr (Fig. 2). MABB was adopted to transfer the HT trait into PB 1121 from Robin. F_1_ plants obtained from the cross, PB 1121 and Robin were confirmed for their hybridity using *AHAS* gene linked SSR marker, RM6844 (Additional file 3: Figure S3). A true F_1_ plant was backcrossed to PB 1121 and 262 BC_1_F_1_ plants were generated. Of these, 128 plants were heterozygous for RM6844 with RPG recovery ranging from 70.98–80.35%. A plant, Pusa 1979–14 with the highest RPG (80.35%) and with a higher level of phenotypic similarity to PB 1121 was backcrossed and 188 BC_2_F_1_ plants were generated, of which 94 plants were heterozygous for foreground marker, RM6844 and the RPG recovery ranged from 81.25 to 92.85%. The superior BC_2_F_1_ plant was further backcrossed and 152 BC_3_F_1_ seeds were produced, out of which 73 plants were found to be heterozygous for the foreground marker and these plants were subjected to phenotypic evaluation for agro-morphological traits and background selection was done using 16 SSR markers remained heterozygous in the BC_2_F_1_ generation. The RPG recovery in the BC_3_F_1_ generation ranged from 93.30 to 95.08%. One superior BC_3_F_1_ plant, with maximum RPG (95.08%) and relatively superior recovery for RPP was backcrossed to generate 122 BC_4_F_1_ plants. A total of 58 plants were found to be heterozygous for RM6844 with RPG recovery range from 94.64 to 98.21%. A plant with maximum RPG recovery (98.21%) was selfed to produce BC_4_F_2_ population. Out of 384 BC_4_F_2_ plants, 88 plants were found to be homozygous for *AHAS* gene linked SSR marker RM6844 (Additional file 4: Figure S4).

**Fig. 3:**
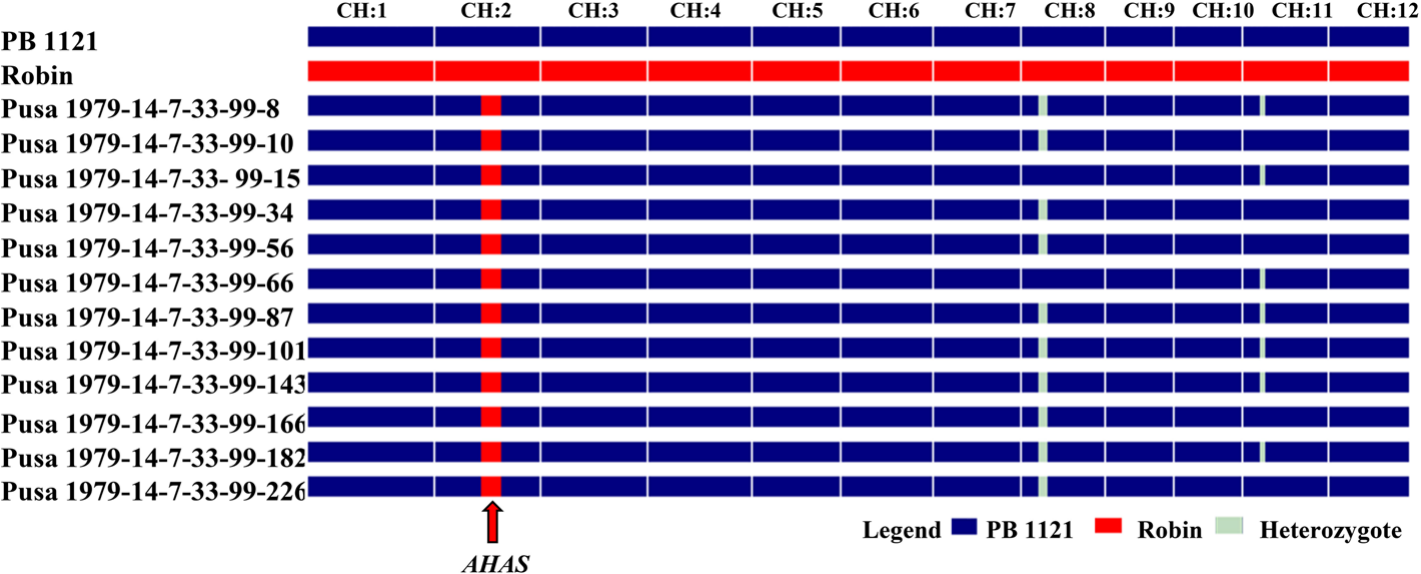
Graphical representation of PB 1121 HT-NILs carrying mutant *AHAS* gene showing the extent of recurrent parent genome (RPG) recovery. CH: chromosome

**Fig. 4:**
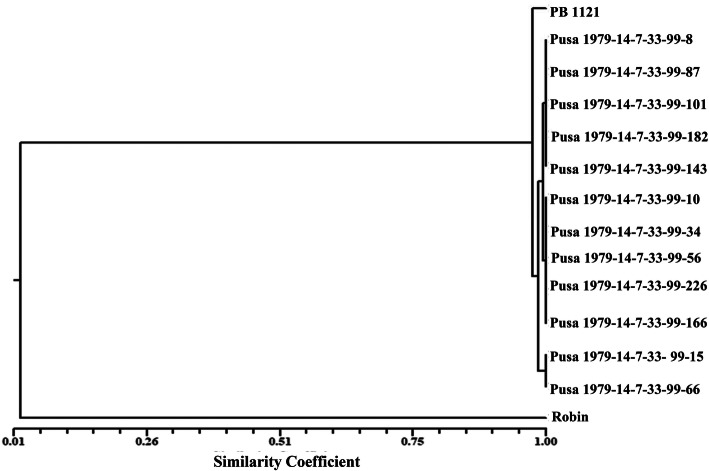
Dendrogram showing the genetic relationship among different PB 1121 HT-NILs in BC_4_F_4_ generation and their parents, PB 1121 and Robin based on 112 polymorphic SSR markers

The mutant *AHAS* homozygous plants were evaluated for grain and cooking quality traits and 40 superior plants were identified. These 40 progenies were further evaluated for yield and quality traits. Based on the family bulk yield and quality traits, twelve families were selected for further detailed evaluation (Table 4). The RPG recovery of these families ranged from 98.66 to 99.55% with an average 99.10%, some residual donor segments were observed in chromosome 8 and 11, whereas complete recovery was achieved in all other chromosomes (Fig. 3). All the PB 1121 HT-NILs clustered together with PB 1121 with an average similarity index (SI) of 0.9756. Among the HT-NILs, Pusa 1979-14-7-33-99-15 and Pusa 1979-14-7-33-99-66 showed maximum similarity with SI of 0.982 (Fig. 4).

**Table 4:** Number of plants generated and recurrent parent genome recovery in the backcross generations during marker aided introgression of mutant *AHAS* allele in PB 1121

Generation	No. of plants generated	No. of plants carrying mutant ***AHAS*** allele in heterozygous/ homozygous state	No. of selected plants/families	Genome recovery (%)	Mean Observed RPG Recovery (%)	Expected Average Recovery (%)
F_1_	15	15^b^	1	^a^		
BC_1_F_1_	262	128^b^	1	70.98–80.35	75.66	75
BC_2_F_1_	188	94^b^	1	81.25–92.85	87.05	87.5
BC_3_F_1_	152	73^b^	1	93.30–95.08	94.19	93.8
BC_4_F_1_	122	58^b^	1	94.64–98.21	96.42	96.9
BC_4_F_2_	384	88^c^	40	^a^	–	–
BC_4_F_3_	40	40^c^	12	^a^	–	–
BC_4_F_4_	12	12^c^	12	98.66–99.55	99.10	–

^a^Not estimated

^b^Plants with mutant *AHAS* allele in heterozygous state

^c^Plants homozygous for mutant *AHAS* allele

**Fig. 5:**
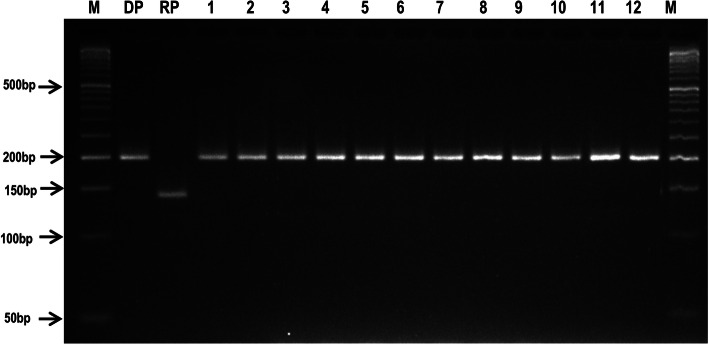
A representative gel image showing the amplification profile of *AHAS* gene linked marker RM6844 in the PB 1121 HT-NILs along with recurrent parent (RP), PB 1121 and donor parent (DP) Robin. M: 50 base pair DNA ladder, DP: Robin, RP: PB 1121, 1–12: PB 1121 HT-NILs, Pusa 1979-14-7-33-99-8, Pusa 1979-14-7-33-99-10, Pusa 1979-14-7-33- 99-15, Pusa 1979-14-7-33-99-34, Pusa 1979-14-7-33-99-56, Pusa 1979-14-7-33-99-66, Pusa 1979-14-7-33-99-87, Pusa 1979-14-7-33-99-101, Pusa 1979-14-7-33-99-143, Pusa 1979-14-7-33-99-166, Pusa 1979-14-7-33-99-182 and Pusa 1979-14-7-33-99-226

Finally, selected 12 BC_4_F_4_ families were validated for homozygosity for the mutated *AHAS* allele using gene linked SSR marker RM6844 (Fig. 5), and also evaluated for Imazethapyr tolerance, agronomic, grain and cooking quality characters in the sprayed and unsprayed condition in a replicated trial.

### Evaluation of PB 1121- HT NILs for Tolerance to Herbicide Imazethapyr

**Fig. 6:**
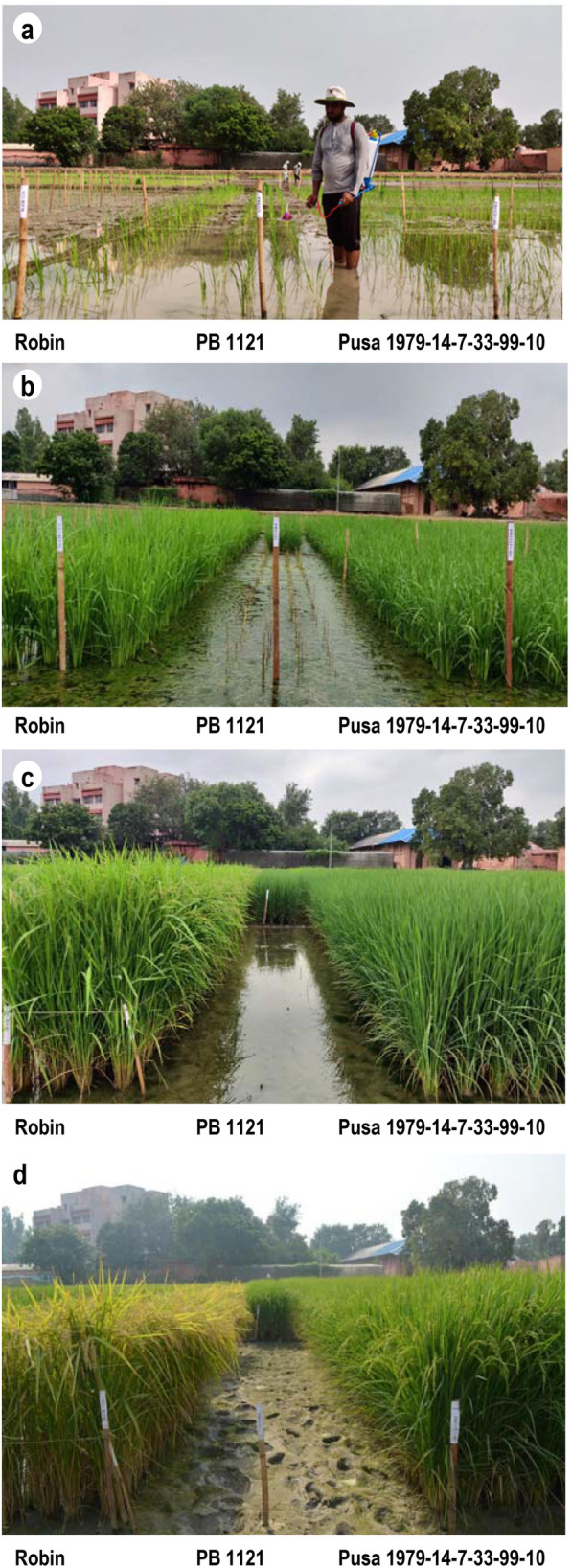
Phenotypic screening of PB 1121-HT NILs along with Recurrent Parent (RP), PB 1121 and Donor Parent, Robin for tolerance to the herbicide, Imazethapyr, **a** application of Imazethapyr @ 2.5 ml/liter after 10 days of transplanting, **b** 20 days after herbicide spray showing that complete death of RP, **c** 60 days after herbicide spray, **d** 90 days after herbicide spray

The PB 1121 HT-NILs along with parents, PB 1121 and Robin were screened for tolerance to herbicide Imazethapyr**.** All the NILs homozygous for the mutant *AHAS* allele exhibited complete tolerance to Imazethapyr. The level of herbicide tolerance in NILs was comparable to DP Robin. While, the RP PB 1121 possessing the wild type *AHAS* allele did not survive after herbicide spray and showed complete mortality within 15 to 20 days of the Imazethapyr spray (Fig. 6).

### Evaluation of PB 1121 HT-NILs for Agronomic, Grain and Cooking Quality

**Fig. 7:**
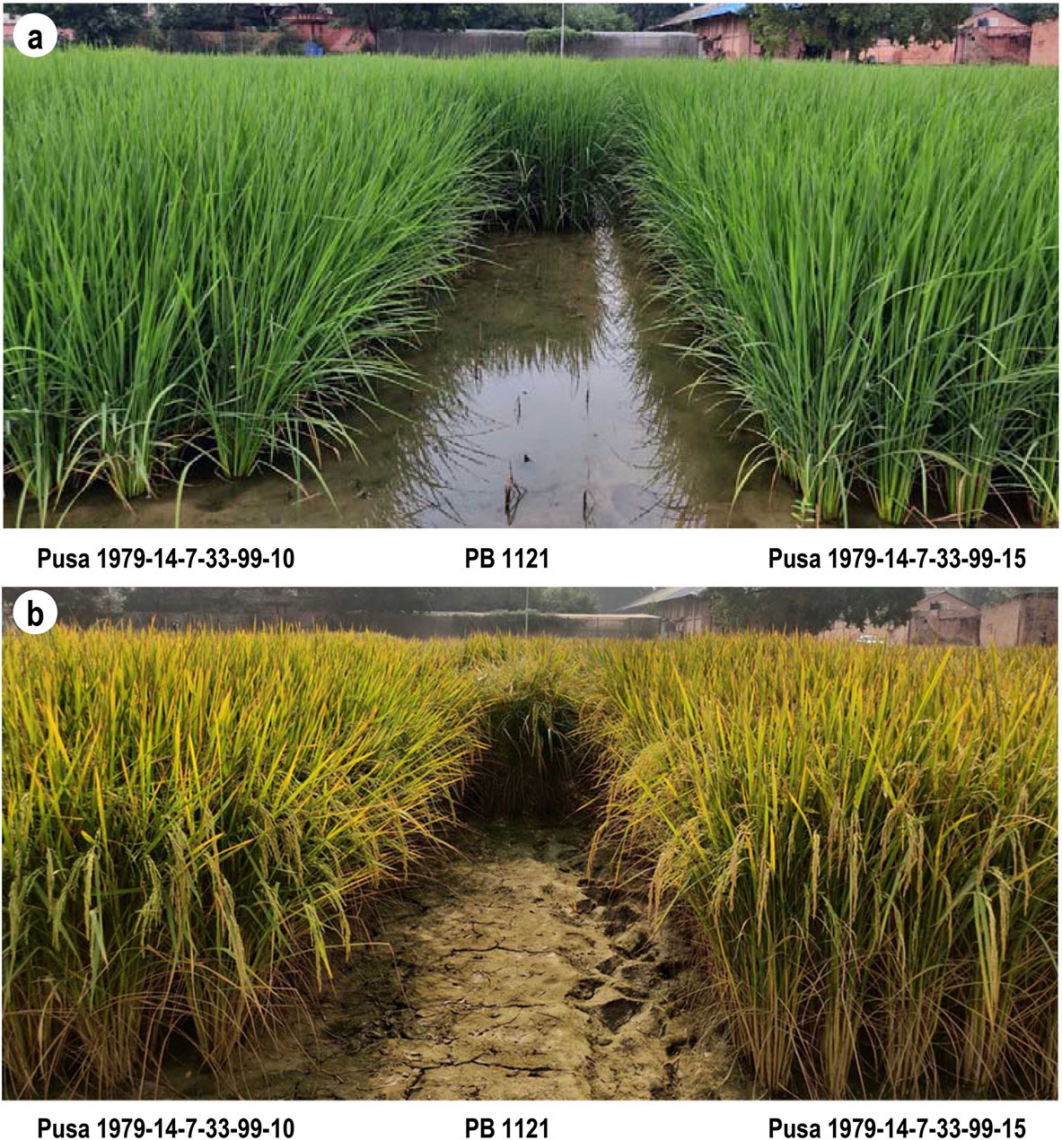
Field view of the PB 1121 HT-NILs at (**a**) booting and (**b**) maturity

**Fig. 8:**
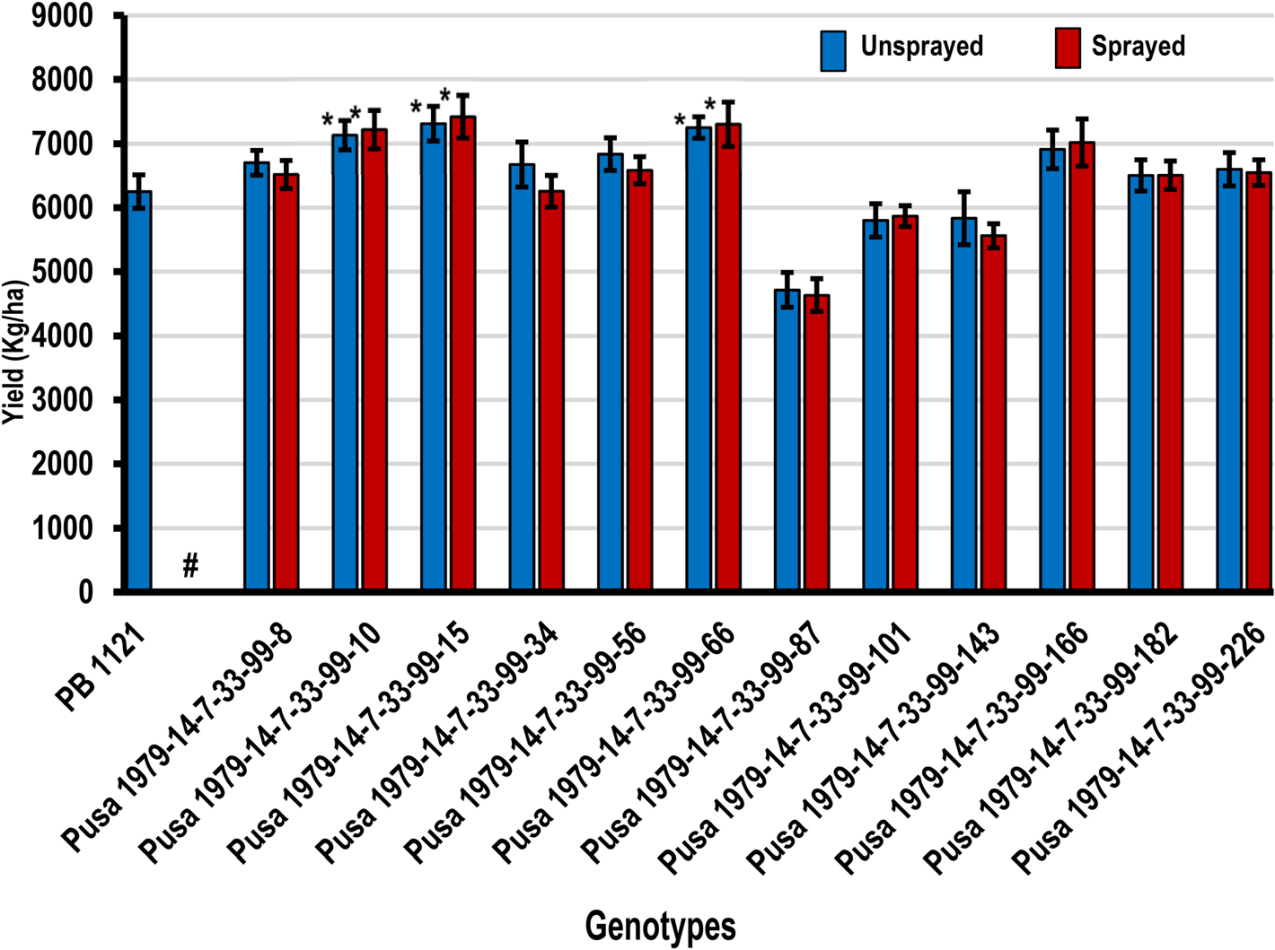
Comparison of yield (kg/ha) between PB 1121 HT-NILs and their parents in unsprayed and herbicide sprayed condition; #PB 1121 seedling did not survive Imazethapyr spray; *Significantly superior to the recurrent parent, PB 1121

The mean performance of PB 1121 HT-NILs for yield, yield components and phenological traits, when grown under herbicide sprayed and unsprayed conditions were recorded. All the data from the sprayed condition and only yield data for unsprayed conditions are presented in Table 5. Under herbicide sprayed conditions, PB 1121 showed complete mortality within 15–20 days of spray, while the NILs showed normal growth and development (Fig. 7). Therefore, the yield and quality traits of NILs under sprayed condition were compared with the data of PB 1121 from unsprayed conditions. It was observed that NILs were at par with PB 1121 in yield, grain and cooking quality traits. NILs Pusa 1979-14-7-33-99-66 and Pusa 1979-14-7-33-99-143 were slightly taller in stature and the NILs Pusa 1979-14-7-33-99-15, Pusa 1979-14-7-33-99-10 and Pusa 1979-14-7-33-99-66 were significantly superior in yield as compared to PB 1121 (Fig. 8 & Table 5) which could be attributed to its higher spikelet fertility. There was no significant difference in the performance of NILs when compared under sprayed and unsprayed conditions (based on Student t-test) while PB 1121 was completely dead under sprayed condition.

**Table 5:** Agronomic performance of PB 1121 HT-NILs in comparison with the recurrent parent PB 1121

Genotype	DFF	PH	NPT	PL	SF%	TGW	YLD (kg/ha)		%RPG
							USP	SP	
Pusa 1979-14-7-33-99-8	106.0	123.15	19.30	27.89	88.20	28.87	6701 ± 274.40	6517 ± 311.34	98.66
Pusa 1979-14-7-33-99-10	109.0^*^	122.45	20.60^*^	28.40	92.70^*^	28.73	7129 ± 322.95^*^	7217 ± 425.32^*^	99.11
Pusa 1979-14-7-33- 99-15	106.0	131.53	20.20^*^	28.52	92.87^*^	28.93	7310 ± 382.05^*^	7417 ± 467.53^*^	99.55
Pusa 1979-14-7-33-99-34	107.0	126.25	19.70^*^	29.28	87.53	27.99	6673 ± 497.09	6255 ± 350.39	99.11
Pusa 1979-14-7-33-99-56	106.5	128.85	17.20	27.56	87.75	28.88	6836 ± 359.89	6582 ± 300.78	99.11
Pusa 1979-14-7-33-99-66	106.5	136.85^*^	19.10^*^	29.29	92.74^*^	28.32	7248 ± 237.14^*^	7300 ± 490.75^*^	99.55
Pusa 1979-14-7-33-99-87	105.5	127.95	15.70	28.79	71.48	26.43	4716 ± 379.94	4634 ± 358.83	98.66
Pusa 1979-14-7-33-99-101	105.5	125.10	13.80	27.58	86.64	27.32	5799 ± 368.33	5866 ± 231.13	98.66
Pusa 1979-14-7-33-99-143	106.0	134.35^*^	15.50	28.56	84.80	28.30	5832 ± 586.79	5559 ± 265.96	98.66
Pusa 1979-14-7-33-99-166	107.0	133.47	17.30	27.73	89.11	28.54	6910 ± 426.37	7017 ± 522.41	99.11
Pusa 1979-14-7-33-99-182	107.5	124.85	16.90	27.33	87.11	27.92	6502 ± 346.16	6506 ± 316.17	98.66
Pusa 1979-14-7-33-99-226	106.0	126.00	16.80	27.48	88.14	27.90	6599 ± 368.68	6547 ± 282.89	99.11
PB 1121^a^	106.0	125.58	16.06	29.81	86.10	28.92	6250 ± 369.38	–	–
**CD (0.05)**	**2.90**	**7.39**	**2.76**	**1.31**	**5.35**	**1.29**	**868.26**	**828.96**	**–**

Data on DFF, PH, NPT, PL, SF%, TGW presented is from Imazethapyr sprayed plots while the yield data (YLD) is presented for both unsprayed and herbicide sprayed plots. *Significant at 5%; *DFF* days to 50% flowering, *PH* Plant height in cm, *NPT* Number of productive tillers, *PL* Panicle length in cm, *SF* Spikelet fertility percentage, *TGW* Thousand grain weight in grams (g), *YLD* Plot yield in kg/ha, *USP* Unsprayed condition, *SP* herbicide (Imazethapyr) Sprayed condition, *RPG* Recurrent Parent Genome, *CD* Critical difference. ^a^Data on DFF, PH, NPT, PL, SF%, TGW for PB 1121 is from unsprayed plots for comparison, as PB 1121 did not survive under Imazethapyr spray

**Fig. 9:**
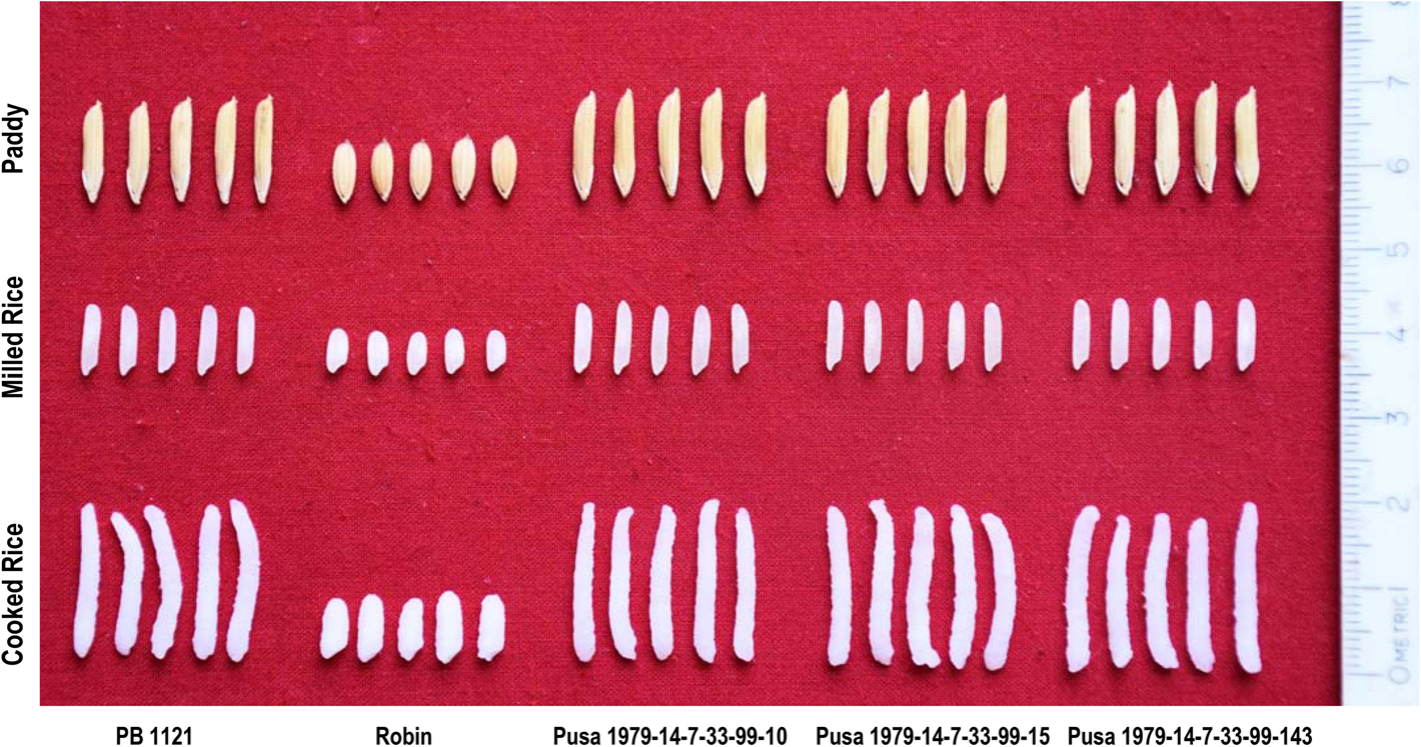
Grain and cooking quality of selected PB 1121 HT-NILs and their parents, PB 1121 and Robin

The mean performance of grain and cooking quality traits of the PB 1121 HT-NILs evaluated under sprayed condition is presented in Table 6. All the NILs possessed extra-long slender grain type with a very strong aroma, and ASV of 7.0, which was similar to that of PB 1121. HUL%l, MIL%, HRR%, KLBC, KBBC, and KBAC were comparable to PB 1121. Three NILs namely, Pusa 1979-14-7-33-99-15, Pusa 1979-14-7-33-99-10 and Pusa 1979-14-7-33-99-143 were found significantly superior for KLAC and elongation ratio as compared to PB 1121 (Table 6 & Fig. 9).

**Table 6:** Grain quality traits of PB 1121 HT-NILs in comparison to PB 1121

Genotype	HUL	MIL	HRR	KLBC	KBBC	KLAC	KBAC	E/R	ASV	AROMA
Pusa 1979-14-7-33-99-8	77.77	67.46	55.96	8.77	1.61	18.54	2.28	2.11	7	3
Pusa 1979-14-7-33-99-10	79.77	70.40	58.40	8.56	1.69	19.66^*^	2.36	2.29^*^	7	3
Pusa 1979-14-7-33- 99-15	78.92	68.33	54.83	8.71	1.68	19.69^*^	2.29	2.26^*^	7	3
Pusa 1979-14-7-33-99-34	76.70	66.73	55.13	8.83	1.67	19.35	2.32	2.18	7	3
Pusa1979-14-7-33-99-56	78.66	67.90	53.80	8.81	1.64	19.10	2.31	2.16	7	3
Pusa 1979-14-7-33-99-66	78.23	68.33	56.05	8.72	1.63	18.97	2.48^*^	2.18	7	3
Pusa 1979-14-7-33-99-87	77.50	69.23	55.36	8.62	1.69	18.46	2.31	2.14	7	3
Pusa 1979-14-7-33-99-101	81.12	70.25	57.15	8.82	1.67	19.32	2.33	2.19	7	3
Pusa 1979-14-7-33-99-143	78.60	69.65	56.67	8.97	1.74	20.29^*^	2.32	2.26^*^	7	3
Pusa 1979-14-7-33-99-166	77.07	67.89	54.09	8.75	1.67	18.88	2.31	2.16	7	3
Pusa 1979-14-7-33-99-182	77.76	66.64	54.95	8.73	1.64	19.32	2.31	2.21	7	3
Pusa 1979-14-7-33-99-226	77.45	67.63	55.42	8.68	1.69	19.04	2.35	2.19	7	3
PB 1121^a^	78.15	69.70	55.80	8.76	1.68	18.82	2.30	2.15	7	3
**CD (0.05)**	**3.41**	**3.99**	**3.27**	**0.31**	**0.09**	**0.78**	**0.08**	**0.08**	–	–

^*^Significant at 5%; *HUL* hulling recovery in percentage, *MIL* milling recovery in percentage, *HRR* head rice recovery in percentage, *KLBC* kernel length before cooking in mm, *KBBC* kernel breadth before cooking in mm, *KLAC* kernel length after cooking in mm, *KBAC* kernel breadth after cooking in mm, *E/R* kernel elongation ratio, *ASV* alkali spreading value, *AROMA* aroma score from panel test; ^a^PB 1121 showed complete mortality after herbicide spray, the data of PB 1121 taken from the unsprayed area is presented for the purpose of comparison

**Fig. 10:**
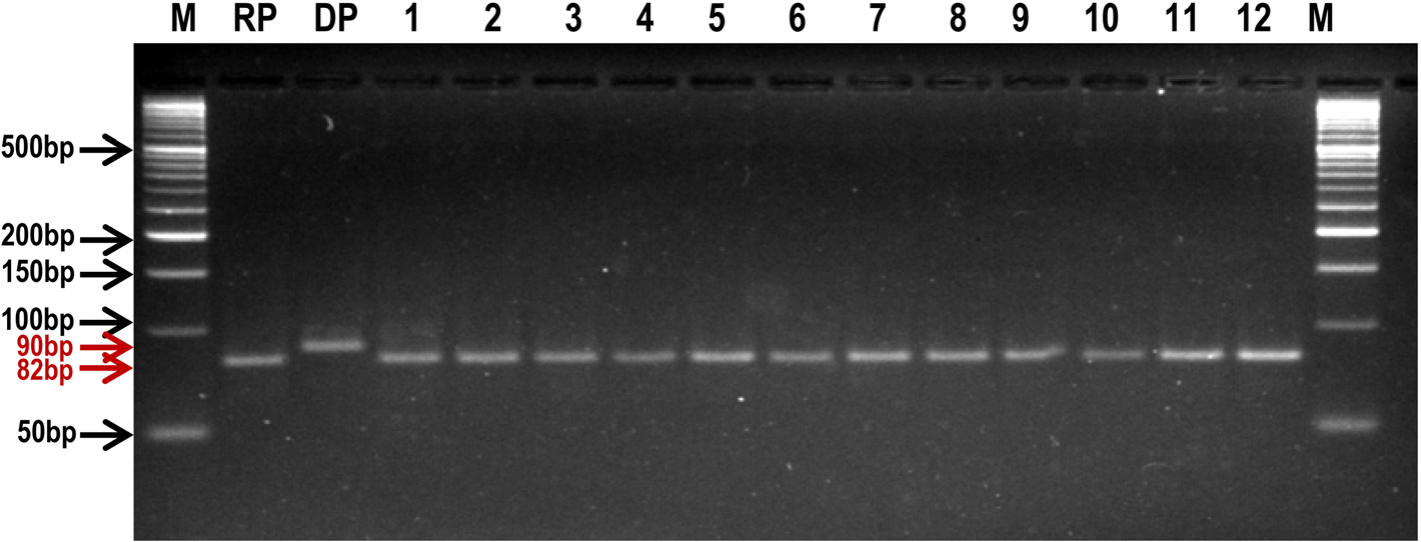
A representative image showing the amplification profile of the marker, *“*nksbad2*”* based on 8 bp deletion in *badh2* in the PB 1121 HT-NILs along with recurrent parent (RP), PB 1121 and the non-aromatic donor parent (DP) Robin. M: 50 base pair DNA ladder, RP: PB 1121, DP: Robin, 1–12: PB 1121 HT-NILs, Pusa 1979-14-7-33-99-8, Pusa 1979-14-7-33-99-10, Pusa 1979-14-7-33- 99-15, Pusa 1979-14-7-33-99-34, Pusa 1979-14-7-33-99-56, Pusa 1979-14-7-33-99-66, Pusa 1979-14-7-33-99-87, Pusa 1979-14-7-33-99-101, Pusa 1979-14-7-33-99-143, Pusa 1979-14-7-33-99-166, Pusa 1979-14-7-33-99-182 and Pusa 1979-14-7-33-99-226

Based on molecular analysis of *badh2* locus, all the HT-NILs were confirmed to have *badh2* allele with 8 bp deletion which is identical to PB 1121 (Fig. 10). In sensory evaluation, the HT-NILs were found to be strongly scented with an aroma score of 3.

## Discussion

Basmati rice of India attracts consumers worldwide due to its peerless grain and cooking quality characteristics and appealing aroma. Among the Basmati rice varieties developed in India, PB 1121 is considered as the hallmark of Basmati, owing to its exceptional KLAC (20 to 25 mm) and high volume expansion upon cooking (> 4 times) (Singh et al. 2018b). Basmati rice is protected under Geographical Indications (GI) for cultivation in seven states of north-western India where rice is primarily cultivated under transplanted conditions. However, due to the limited availability of labor on time, high transplanting cost and depleting water table, there is a need to shift rice cultivation to DSR. Although DSR is a potential technology, the response of farmers for its adoption has been lukewarm owing to heavy weed infestation under DSR. Herbicide tolerant rice varieties provide a potential alternative for effective weed management under DSR.

MABB has several advantages over conventional breeding and has been successfully deployed for improving elite rice varieties for resistance to biotic stresses such as bacterial blight and blast (Joseph et al. 2004; Gopalakrishnan et al. 2008; Singh et al. 2012a, 2012b; Singh et al. 2013; Khanna et al. 2015; Ellur et al. 2016a, 2016b), and abiotic stresses such as salinity tolerance (Babu et al. 2017; Singh et al. 2018a) which it has brought a paradigm shift in Basmati rice breeding (Singh et al. 2011).

In this study, we report the transfer of herbicide tolerance trait from Robin into the genetic background of PB 1121 through MABB. Robin is an EMS induced mutant of an upland drought tolerant rice variety N22, which possess tall plant stature, short grains and lacks aroma (Shoba et al. 2017). Using poor grain quality DP to transfer HT trait into the genetic background of high quality Basmati rice variety PB 1121, is a challenging task especially for the recovery of exquisite grain and cooking quality of this Basmati rice variety in the improved HT-NILs. Foreground selection together with background and phenotypic selection led to precise transfer of mutant *AHAS* allele for herbicide tolerance as well as accelerated the RPG recovery to an extent of 98.66 to 99.55% with four backcross generations. The complete recovery of RPG in mutant *AHAS* gene carrier chromosome as well as rest of chromosome was achieved except chromosomes 8 and 11, which retained some residual donor fragment (Fig. 3). Using the SSR markers for background analysis generally target the non-coding and heterochromatic regions and hence could not quantify the recovery of functional part of the genome. Therefore, background selection was coupled with phenotypic selection for RPP which helped in speeding the process of reconstruction of RPP as it entails the indirect selection for functionally expressed part of the genome (Ellur et al. 2016a).

PB 1121 HT-NILs were at par with PB 1121 for agro-morphological, grain and cooking quality traits, while exhibiting herbicide tolerance similar to Robin. This was possible due to stringent phenotypic selection for agro-morphological traits, grain and cooking quality traits for RP PB 1121 phenotype carried out in each of the backcross generations. The significance of phenotypic selection with background selection in the development of NILs with maximum RPG and RPP recovery has also been earlier demonstrated (Ellur et al. 2016b; Babu et al. 2017). Three NILs, Pusa 1979-14-7-33-99-15, Pusa 1979-14-7-33-99-10 and Pusa 1979-14-7-33-99-66 were significantly superior in yield, grain and cooking quality as compared to PB 1121 indicating the effectiveness of augmenting rigorous phenotypic selection for the cooking quality traits along with background selection.

Pleasant aroma is an essential trait of Basmati rice, which is primarily governed by a recessive gene *badh2* located on chromosome 8. The accumulation of 2-acetyl-1-pyrroline (2-AP) in aromatic rice is explained by the loss of function mutations in the *badh2* gene (Bradbury et al. 2005; Chen et al. 2008). All the PB 1121 HT-NILs and the recurrent parent PB 1121 were found to carry an 8 bp deletion corresponding to the aromatic allele for the production of 2-AP (Fig. 10).

AHAS is an enzyme which catalyzes two reactions for the synthesis of branched chain amino acids namely, valine, leucine, and isoleucine (Singh and Shaner 1995). Condensation of two pyruvate molecules forms 2-acetolactate which leads to the formation of valine and leucine. While for isoleucine biosynthesis, 2-acetohydroxybutyrate is synthesized from pyruvate and 2-ketobutyrate (Zhou et al. 2007). AHAS-inhibiting herbicides block the substrate access channel in the AHAS enzyme by binding to it and elicit deficiency of branched-chain amino acids (Garcia et al. 2017). The resulting decrease of protein synthesis slows down cell division thus leading to growth difficulties in plants (Yu and Powles 2014). However, the mutations within the *AHAS* gene results in altered AHAS enzyme which confers resistance to the AHAS inhibiting herbicides (Tranel and Wright 2002; Duggleby and Pang 2000; Christoffers et al. 2006). All the PB 1121 HT-NILs carrying mutated *AHAS* allele displayed tolerance to Imazethapyr herbicide when applied @ 2.5 ml/liter with no significant difference in the yield performance, grain and cooking quality traits when compared to HT-NILs under unsprayed conditions. This indicates the effectiveness of mutant *AHAS* gene to overcome the adverse effect of herbicide Imazethapyr.

In all, the present study reports successful development of HT NILs in the genetic background of popular Basmati rice variety PB 1121 by introgression of mutated *AHAS* allele using MABB program. The PB 1121 HT-NILs are being evaluated in the National Basmati Trials for their subsequent release as commercial varieties. These NILs will help the farmers in adopting DSR in Basmati rice production which would help in economizing the rice production.

## Supplementary information

**Additional file 1: Figure S1.** Agarose gel image (a-e) of all 112 SSR markers polymorphic between RP PB 1121 and DP Robin. M: 50 base pair DNA ladder; P1: PB 1121; P2: Robin.

**Additional file 2: Figure S2.** Chromosome wise Graphical representation of all 112 SSR markers polymorphic between RP PB 1121 and DP Robin.

**Additional file 3: Figure S3.** Gel image showing the amplification profile of *AHAS* linked SSR marker RM6844 in the F_1_ plants. M: 50 base pair DNA ladder; DP: Robin; RP: PB 1121; 1–15: F_1_ plants.

**Additional file 4: Figure S4.** A representative gel amplification image of the SSR marker, RM6844 used in foreground selection in the BC_4_F_2_ population. M: 50 base pair DNA ladder; RP: PB 1121; DP: Robin; 1–68: BC_4_F_2_ plants.

**Additional file 5: Table S1.** Details of 112 polymorphic SSR markers used in Background selection for development of PB 1121 HT-NILs.

## Data Availability

All relevant data are provided as Tables within the paper and in the Supporting Information files.

## Abbreviations

DSR: Direct-seeded rice
HT: Herbicide tolerant
MABB: Marker assisted backcross breeding
*AHAS*: Acetohydroxy acid synthase
PB: Pusa Basmati
DP: Donor parent
RP: Recurrent parent
NIL: Near-isogenic line
RPP: Recurrent parent phenome
RPG: Recurrent parent genome
SSR: Simple sequence repeat
*ALS*: Acetolactate synthase
EMS: Ethyl methane sulfonate
2-AP: 2-acetyl-1-pyrroline
CD: Critical difference
