# Supplementary figures and images for: Marker Assisted Development and Characterization of Herbicide Tolerant Near Isogenic Lines of a Mega Basmati Rice Variety, “Pusa Basmati 1121”
Source: Rice (N Y). 2020 Sep 15;13:68. doi: 10.1186/s12284-020-00423-2 (PMC7492307; doi:10.1186/s12284-020-00423-2)

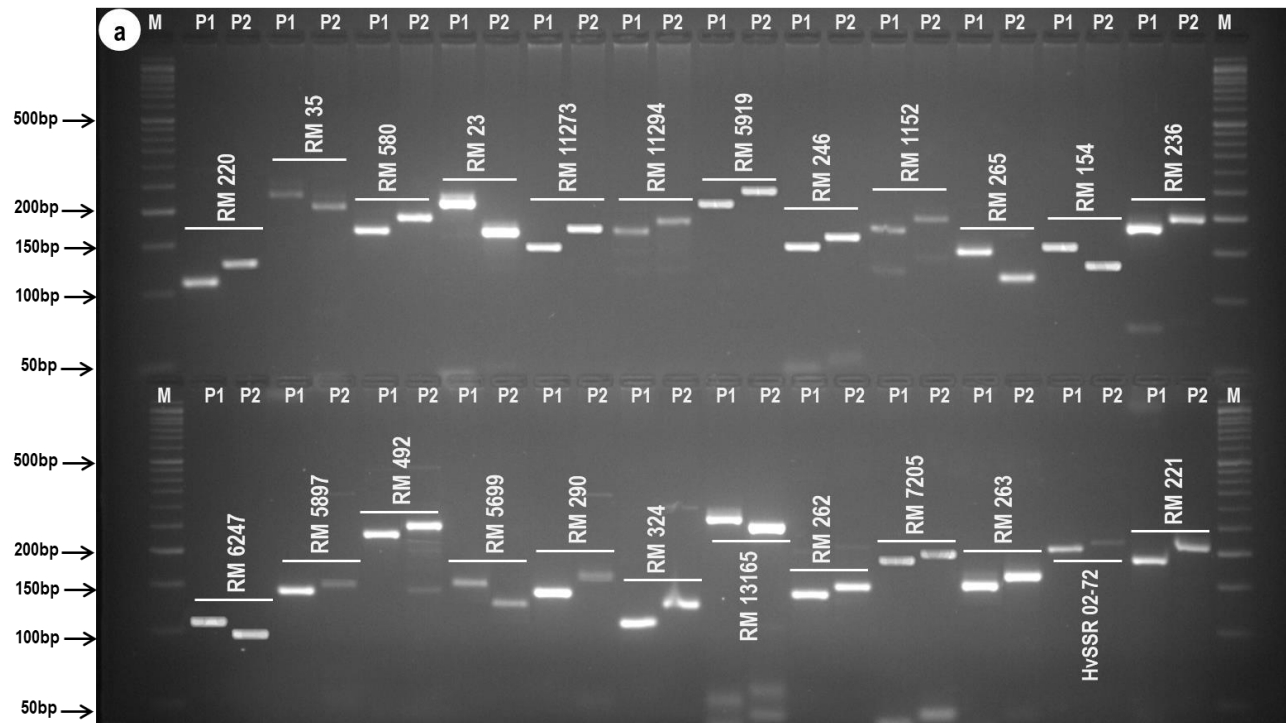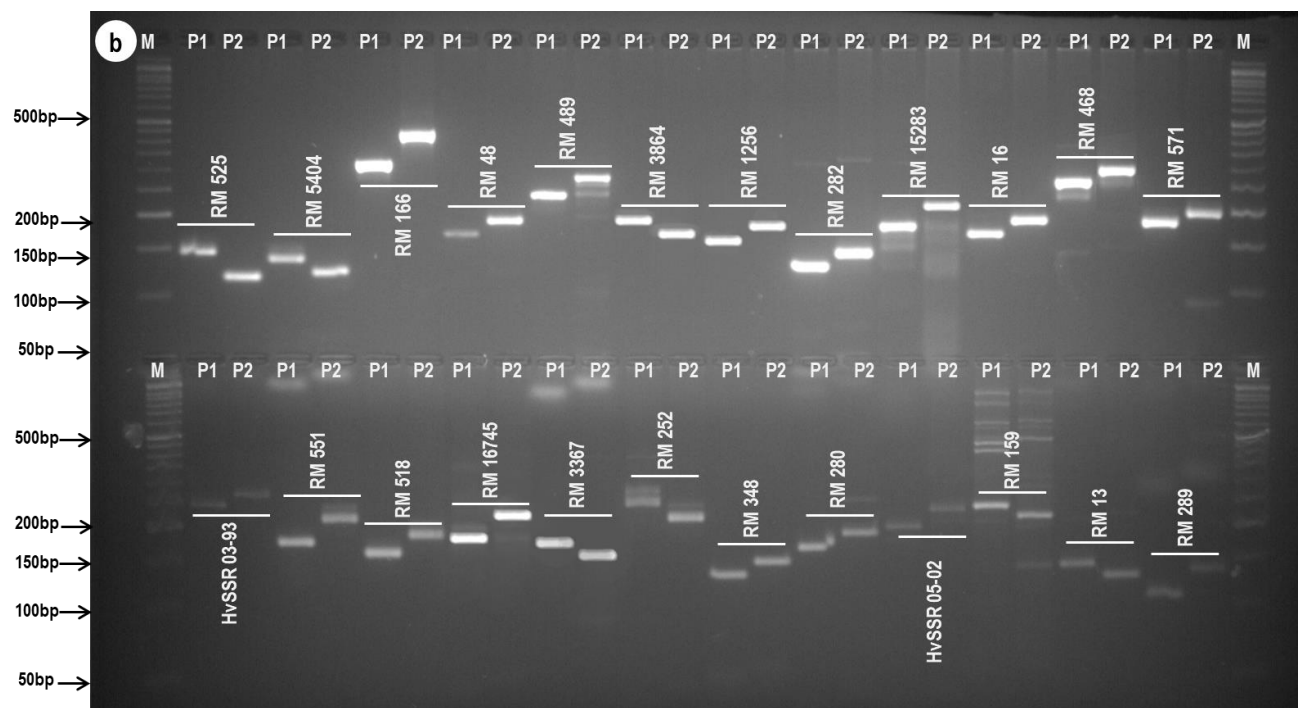

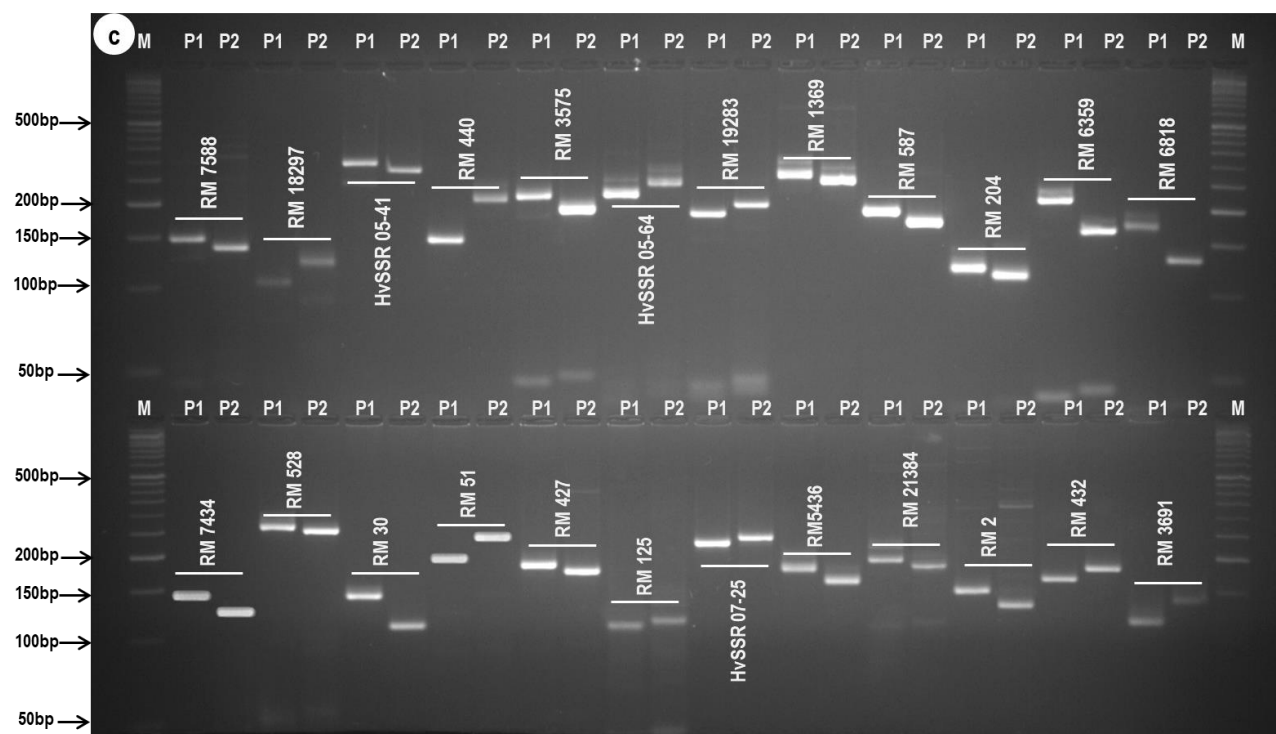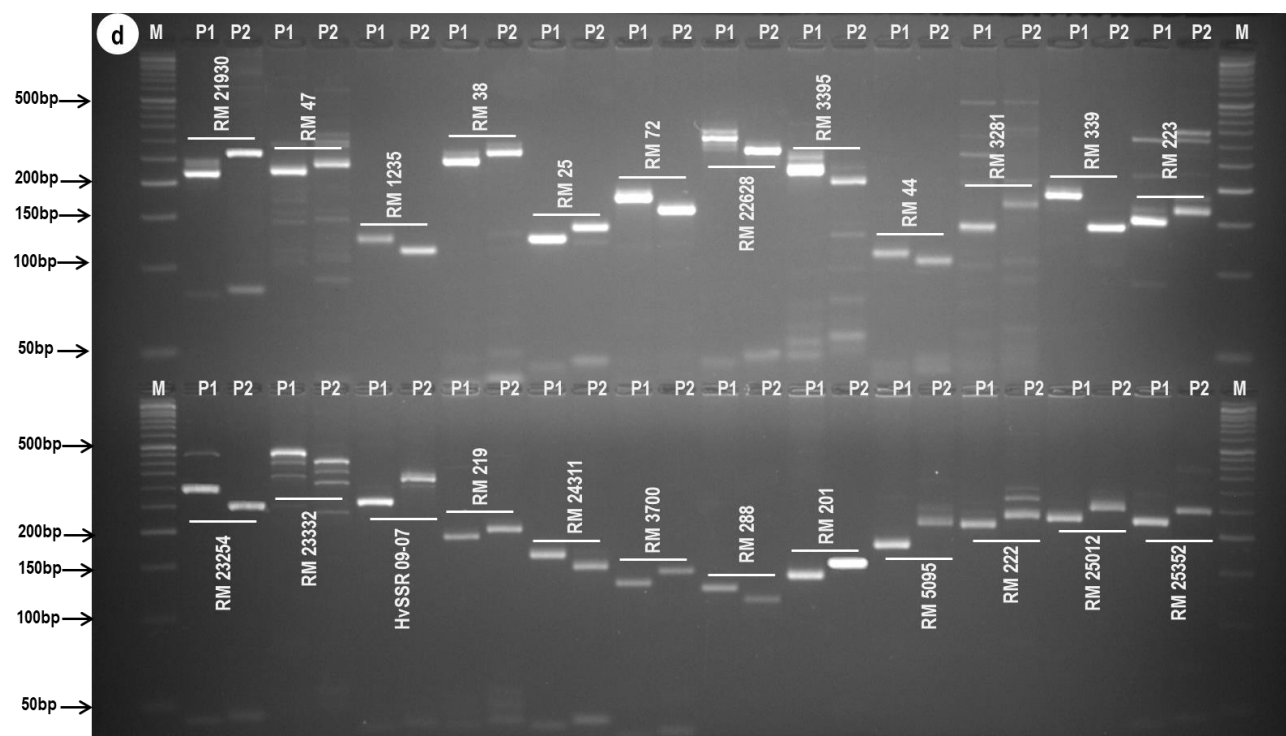

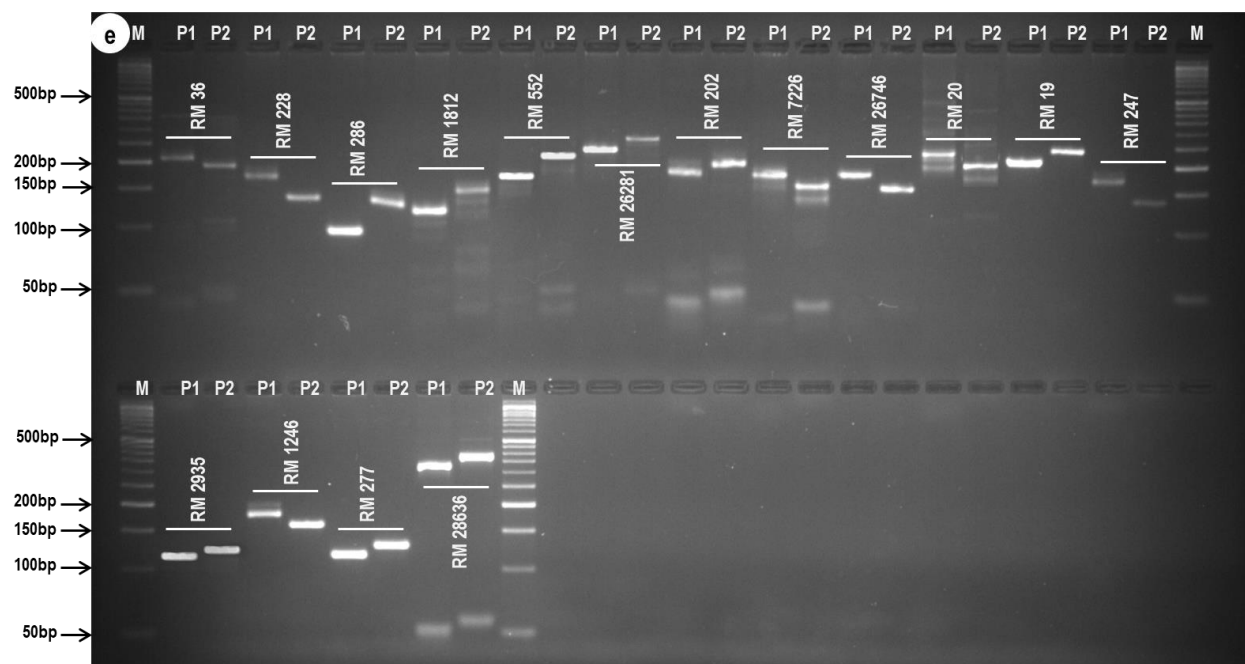

Supplement: Supplementary file 1 — Additional file 1: Figure S1. Agarose gel image (a-e) of all 112 SSR markers polymorphic between RP PB 1121 and DP Robin. M: 50 base pair DNA ladder; P1: PB 1121; P2: Robin. [file 12284_2020_423_MOESM1_ESM.pdf]

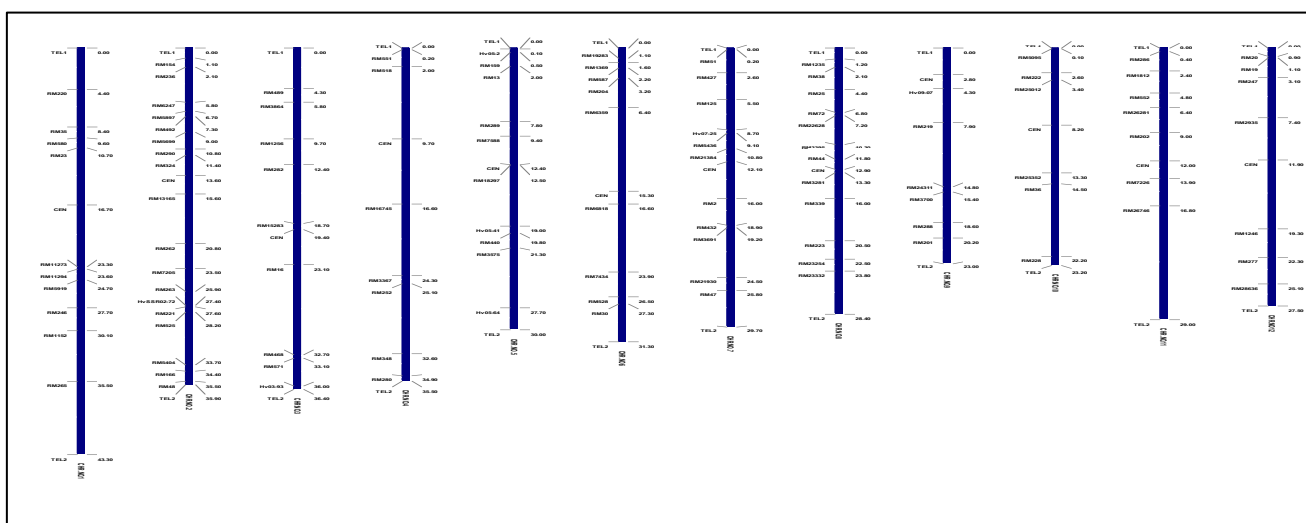

Supplement: Supplementary file 2 — Additional file 2: Figure S2. Chromosome wise Graphical representation of all 112 SSR markers polymorphic between RP PB 1121 and DP Robin. [file 12284_2020_423_MOESM2_ESM.pdf]

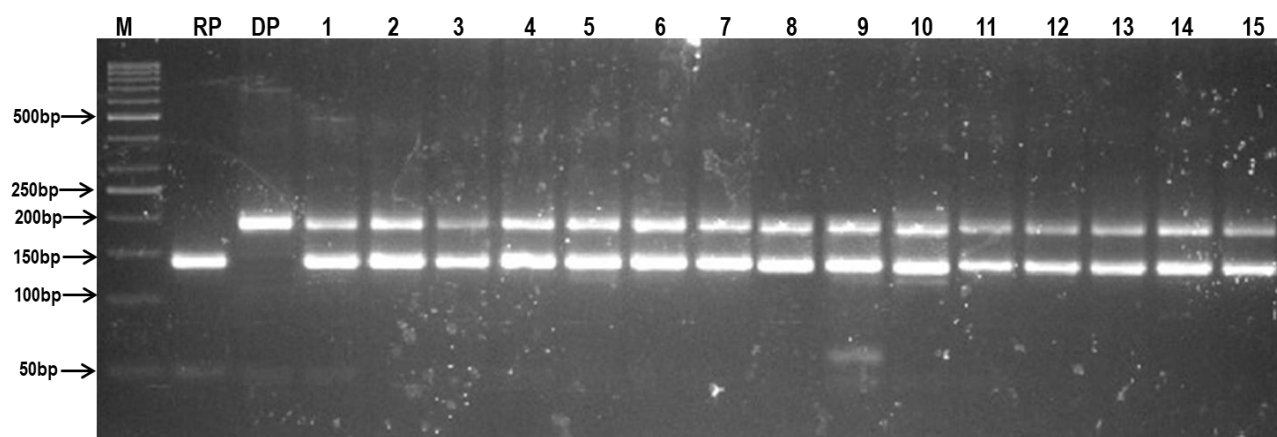

Supplement: Supplementary file 3 — Additional file 3: Figure S3. Gel image showing the amplification profile of AHAS linked SSR marker RM6844 in the F1 plants. M: 50 base pair DNA ladder; DP: Robin; RP: PB 1121; 1–15: F1 plants. [file 12284_2020_423_MOESM3_ESM.pdf]

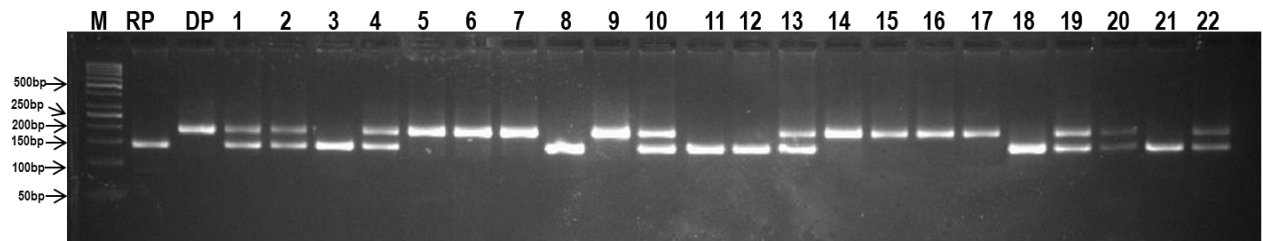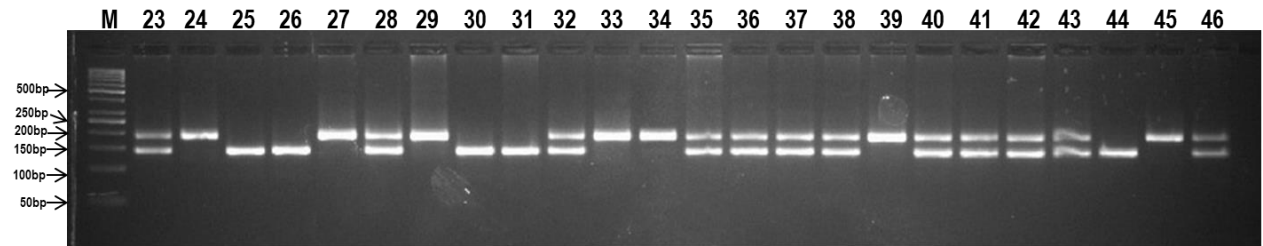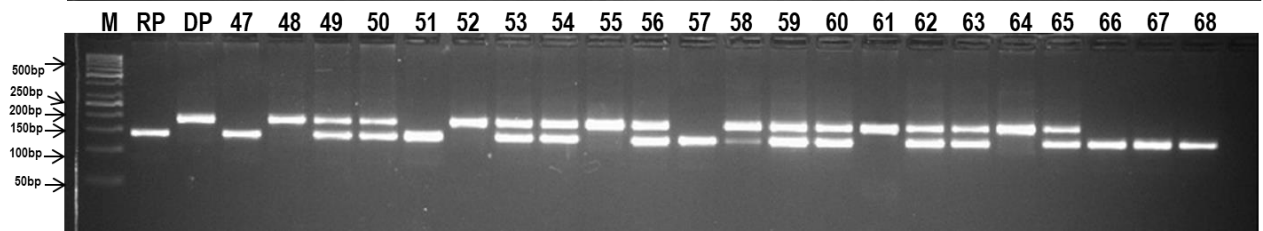

Supplement: Supplementary file 4 — Additional file 4: Figure S4. A representative gel amplification image of the SSR marker, RM6844 used in foreground selection in the BC4F2 population. M: 50 base pair DNA ladder; RP: PB 1121; DP: Robin; 1–68: BC4F2 plants. [file 12284_2020_423_MOESM4_ESM.pdf]
