# Supplementary material for: Marker Assisted Development and Characterization of Herbicide Tolerant Near Isogenic Lines of a Mega Basmati Rice Variety, “Pusa Basmati 1121”
Source: Rice (N Y). 2020 Sep 15;13:68. doi: 10.1186/s12284-020-00423-2 (PMC7492307; doi:10.1186/s12284-020-00423-2)
Supplement: Supplementary file 5 — Additional file 5: Table S1. Details of 112 polymorphic SSR markers used in Background selection for development of PB 1121 HT-NILs. [file 12284_2020_423_MOESM5_ESM.docx]

**Additional file 5: Table S1.** Details of 112 polymorphic SSR markers used in Background selection for development of PB 1121 HT-NILs

| **S. No** | **Markers Name** | **Chromosome** | **Physical Location** | **Forward Primer Sequences** | **Reverse Primer Sequences** | **Tm** |
| --- | --- | --- | --- | --- | --- | --- |
| 1 | RM220 | 1 | 4.42 | GGAAGGTAACTGTTTCCAAC | GAAATGCTTCCCACATGTCT | 55**°C** |
| 2 | RM35 | 1 | 8.40 | TGGTTAATCGATCGGTCGCC | CGACGGCAGATATACACGG | 55**°C** |
| 3 | RM580 | 1 | 9.60 | CGACGGCAGATATACACGG | CACTCCCATGTTTGGCTCC | 55**°C** |
| 4 | RM23 | 1 | 10.67 | CATTGGAGTGGAGGCTGG | GTCAGGCTTCTGCCATTCTC | 55**°C** |
| 5 | RM11273 | 1 | 23.28 | CGATCCCAGCCGTCCATTTCC | CGATCCCAGCCGTCCATTTCC | 55**°C** |
| 6 | RM11294 | 1 | 23.56 | CATCGCTTAGCAATCTCCTTGG | GACAAACGGAGAGGGAAGAGACC | 55**°C** |
| 7 | RM5919 | 1 | 24.70 | AAACAGTCAGCGGCTTTGTC | ATAGCGTTTGACGGGACAAC | 55**°C** |
| 8 | RM246 | 1 | 27.66 | GAGCTCCATCAGCCATTCAG | CTGAGTGCTGCTGCGACT | 55**°C** |
| 9 | RM1152 | 1 | 30.08 | GCCTTTGTCCTTCAGTAGGC | AGAGCGCCTGGGTATAATTG | 55**°C** |
| 10 | RM265 | 1 | 35.52 | CGAGTTCGTCCAAGTGAGC | CATCCACCATTCCACCAATC | 55**°C** |
| 11 | RM154 | 2 | 1.08 | GACGGTGACGCACTTTATGAACC | CGATCTGCGAGAAACCCTCTCC | 55**°C** |
| 12 | RM236 | 2 | 2.11 | GCGCTGGTGGAAAATGAG | GGCATCCCTCTTTGATTCCTC | 55**°C** |
| 13 | RM6247 | 2 | 5.79 | CGCTCTTGTCTTTACTCCCG | GCTGCTGCTGCTTCTTTTTC | 55**°C** |
| 14 | RM5897 | 2 | 6.73 | GGCATCTTCCCCTCTCTCTC | CCAACCCAAACCAGTCTACC | 55**°C** |
| 15 | RM492 | 2 | 7.28 | CCAAAAATAGCGCGAGAGAG | AAGACGTACATGGGTCAGGC | 55**°C** |
| 16 | RM5699 | 2 | 8.98 | ATCGTTTCGCATATGTTT | ATCGGTAAAAGATGAGCC | 55**°C** |
| 17 | RM290 | 2 | 10.8 | ACCCTTATTCCTGCTCTCCTC | GTGCTGTAGATGGAAGGGAG | 55**°C** |
| 18 | RM 324 | 2 | 11.38 | CTGATTCCACACACTTGTGC | GATTCCACGTCAGGATCTTC | 55**°C** |
| 19 | RM13165 | 2 | 15.56 | GTGATCCGCTACCACATTCATTATGC | CCGGAATTTCTTAACACTCTCACACC | 55**°C** |
| 20 | RM 262 | 2 | 20.79 | CATTCCGTCTCGGCTCAACT | CAGAGCAAGGTGGCTTGC | 55**°C** |
| 21 | RM7205 | 2 | 23.5 | TGTGGGAATTTCTAGCATCC | AGGAAGGAGAGAGGTCTGAGG | 55**°C** |
| 22 | RM263 | 2 | 25.86 | CCCAGGCTAGCTCATGAACC | GCTACGTTTGAGCTACCACG | 55**°C** |
| 23 | HvSSR02-72 | 2 | 27.42 | TTTGTGTTGTAACGGATGAA | TATTTGATATCATGGTGCCC | 55**°C** |
| 24 | RM221 | 2 | 27.60 | ACATGTCAGCATGCCACATC | TGCAAGAATCTGACCCGG | 55**°C** |
| 25 | RM525 | 2 | 28.20 | GGCCCGTCCAAGAAATATTG | CGGTGAGACAGAATCCTTACG | 55**°C** |
| 26 | RM5404 | 2 | 33.67 | GGCCATCCATCTCCTGTATG | GACACACACAGGGTTGGTTG | 55**°C** |
| 27 | RM166 | 2 | 34.35 | GGTCCTGGGTCAATAATTGGGTTACC | TTGCTGCATGATCCTAAACCGG | 55**°C** |
| 28 | RM48 | 2 | 35.50 | TGTCCCACTGCTTTCAAGC | CGAGAATGAGGGACAAATAACC | 55**°C** |
| 29 | RM489 | 3 | 4.30 | ACTTGAGACGATCGGACACC | TCACCCATGGATGTTGTCAG | 55**°C** |
| 30 | RM3864 | 3 | 5.85 | AGTCAACCTTGGGGGTAAGG | AGATACTGCCCGTGTCATCC | 55**°C** |
| 31 | RM1256 | 3 | 9.70 | ACGCGAAGCAACGGAGATAG | CTAGCCTCGATGCGAAAAAC | 55**°C** |
| 32 | RM282 | 3 | 12.40 | CTGTGTCGAAAGGCTGCAC | CAGTCCTGTGTTGCAGCAAG | 55**°C** |
| 33 | RM15283 | 3 | 18.71 | GCTACAAATAGCTGCAAACTGC | TTGGACTAGCCTTTGACTGAGG | 55**°C** |
| 34 | RM16 | 3 | 23.08 | CGCTAGGGCAGCATCTAAA | AACACAGCAGGTACGCGC | 55**°C** |
| 35 | RM468 | 3 | 32.67 | CCCTTCCTTGTTGTGGCTAC | TGATTTCTGAGAGCCAACCC | 55**°C** |
| 36 | RM571 | 3 | 33.10 | GGAGGTGAAAGCGAATCATG | CCTGCTGCTCTTTCATCAGC | 55**°C** |
| 37 | Hv03-93 | 3 | 36.00 | GACTGACTTCGGTGTTCATT | TGCGTTCTTATATATGGGCT | 55**°C** |
| 38 | RM551 | 4 | 0.17 | AGCCCAGACTAGCATGATTG | GAAGGCGAGAAGGATCACAG | 55**°C** |
| 39 | RM518 | 4 | 2.03 | CTCTTCACTCACTCACCATGG | ATCCATCTGGAGCAAGCAAC | 55**°C** |
| 40 | RM16745 | 4 | 16.63 | TGAGGAGTGAGGAGAGTGATAAACC | GCATATGGTTTGGTTGATGTCC | 55**°C** |
| 41 | RM3367 | 4 | 24.26 | GGATCCATCCATCCACTGAC | GGATATGTGCTGCTGTGTGC | 55**°C** |
| 42 | RM252 | 4 | 25.14 | TTCGCTGACGTGATAGGTTG | ATGACTTGATCCCGAGAACG | 55**°C** |
| 43 | RM348 | 4 | 32.60 | CCGCTACTAATAGCAGAGAG | GGAGCTTTGTTCTTGCGAAC | 55**°C** |
| 44 | RM280 | 4 | 34.90 | ACACGATCCACTTTGCGC | TGTGTCTTGAGCAGCCAGG | 55**°C** |
| 45 | Hv05-2 | 5 | 0.14 | TATTGGCCATTGATTACTCC | CATCTTACAAACTGAAACGGA | 55**°C** |
| 46 | RM159 | 5 | 0.48 | GGGGCACTGGCAAGGGTGAAGG | GCTTGTGCTTCTCTCTCTCTCTCTCTCTC | 55**°C** |
| 47 | RM13 | 5 | 2.01 | TCCAACATGGCAAGAGAGAG | GGTGGCATTCGATTCCAG | 55**°C** |
| 48 | RM289 | 5 | 7.80 | TTCCATGGCACACAAGCC | CTGTGCACGAACTTCCAAAG | 55**°C** |
| 49 | RM7588 | 5 | 9.40 | CGGGGCTTTCTTTCTTTCTC | AAGCCCATGACGTATCGAAG | 55**°C** |
| 50 | RM18297 | 5 | 12.50 | CGATGGCTTGATGGTTACATCG | CGTCCTGGATTGAATTAAGCTTCG | 55**°C** |
| 51 | Hv05-41 | 5 | 18.99 | CCATTCCCAAATATACCAGA | ACAAGGGTTCCCGATATAAT | 55**°C** |
| 52 | RM440 | 5 | 19.83 | CATGCAACAACGTCACCTTC | ATGGTTGGTAGGCACCAAAG | 55**°C** |
| 53 | RM3575 | 5 | 21.30 | CCTGGAATGATGATGGAAGG | GTTTTGCTTCCTGGAAGTGC | 55**°C** |
| 54 | Hv05-64 | 5 | 27.70 | GAGTACCCTGTTCCACCATA | CACAAACAACAGCATTTCAC | 55**°C** |
| 55 | RM19283 | 6 | 1.07 | AGCCGTGGATGACCTTCATGACC | ACACCACAGCATGCGCAACG | 55**°C** |
| 56 | RM1369 | 6 | 1.56 | AACCTGAGAGTGCCAATTGG | TCCCCTAGTAAAGCGGATTC | 55**°C** |
| 57 | RM587 | 6 | 2.20 | ACGCGAACAAATTAACAGCC | CTTTGCTACCAGTAGATCCAGC | 55**°C** |
| 58 | RM204 | 6 | 3.16 | GTGACTGACTTGGTCATAGGG | GCTAGCCATGCTCTCGTACC | 55**°C** |
| 59 | RM6359 | 6 | 6.44 | ACCGAATCCCACTCTTCCTC | AGGAGGAGGAGGATGAGCTC | 55**°C** |
| 60 | RM6818 | 6 | 16.58 | GTCGCATTCGTCTCCACC | ACCATTTCCAGATGACTCGG | 55**°C** |
| 61 | RM7434 | 6 | 23.93 | GGAGGAAAGGTTGGAGAAGG | TTTCCCGTATTCCATGAGCC | 55**°C** |
| 62 | RM528 | 6 | 26.50 | GGCATCCAATTTTACCCCTC | AAATGGAGCATGGAGGTCAC | 55**°C** |
| 63 | RM30 | 6 | 27.25 | GGTTAGGCATCGTCACGG | TCACCTCACCACACGACACG | 55**°C** |
| 64 | RM51 | 7 | 0.23 | TCTCGATTCAATGTCCTCGG | CTACGTCATCATCGTCTTCCC | 55**°C** |
| 65 | RM427 | 7 | 2.60 | TCACTAGCTCTGCCCTGACC | TGATGAGAGTTGGTTGCGAG | 55**°C** |
| 66 | RM125 | 7 | 5.48 | ATCAGCAGCCATGGCAGCGACC | AGGGGATCATGTGCCGAAGGCC | 55**°C** |
| 67 | Hv07-25 | 7 | 8.66 | CACGTGTACGTGTCTCTGAA | GTCCACAACGATTTCCTTTA | 55**°C** |
| 68 | RM5436 | 7 | 9.07 | CAAAGGGGGTGTCCTCTATG | GTTGCTCGTCCTACATGTGC | 55**°C** |
| 69 | RM21384 | 7 | 10.75 | CTCCTCCCATTGTTCACCACTCC | AGGAGGATGGAAGGGCACTAGC | 55**°C** |
| 70 | RM2 | 7 | 16.02 | ACGTGTCACCGCTTCCT | ATGTCCGGGATCTCATCG | 55**°C** |
| 71 | RM432 | 7 | 18.90 | TTCTGTCTCACGCTGGATTG | AGCTGCGTACGTGATGAATG | 55**°C** |
| 72 | RM3691 | 7 | 19.20 | GCTGATGGTCAAAGATCAGG | ATGTGTCTGCTGGCACAGAG | 55**°C** |
| 73 | RM21930 | 7 | 24.50 | TAGCTGTTGTGCATGATGTTCG | GCTGGACTCCTCTTGATCTCTCC | 55**°C** |
| 74 | RM47 | 7 | 25.80 | ACTCCACTCCACTCCCCAC | GTCAGCAGGTCGGACGTC | 55**°C** |
| 75 | RM1235 | 8 | 1.20 | AGCAGAGGAGGAGATGATGG | GGACCAAAACGAAGCTATCC | 55**°C** |
| 76 | RM38 | 8 | 2.11 | ACGAGCTCTCGATCAGCCTA | TCGGTCTCCATGTCCCAC | 55**°C** |
| 77 | RM25 | 8 | 4.37 | GGAAAGAATGATCTTTTCATGG | CTACCATCAAAACCAATGTTC | 55**°C** |
| 78 | RM72 | 8 | 6.76 | CCGGCGATAAAACAATGAG | GCATCGGTCCTAACTAAGGG | 55**°C** |
| 79 | RM22628 | 8 | 7.22 | GTGATGGACGGTAGATGGAACC | CTCACACACGAGGACTTTATCACG | 55**°C** |
| 80 | RM3395 | 8 | 10.28 | ACCTCATGTCCAGGTGGAAG | AGATTAGTGCCATGGCAAGG | 55**°C** |
| 81 | RM44 | 8 | 11.75 | ACGGGCAATCCGAACAACC | TCGGGAAAACCTACCCTACC | 55**°C** |
| 82 | RM3281 | 8 | 13.30 | TCAATTTTGAAGCCCCCTCC | AAAAATGGAGAGGAGGACGC | 55**°C** |
| 83 | RM339 | 8 | 16.02 | GTAATCGATGCTGTGGGAAG | GAGTCATGTGATAGCCGATATG | 55**°C** |
| 84 | RM223 | 8 | 20.51 | GAGTGAGCTTGGGCTGAAAC | GAAGGCAAGTCTTGGCACTG | 55**°C** |
| 85 | RM23254 | 8 | 22.50 | TTCACTGGTCCTTAGCTTCTGAGC | CACCAACCGGGACTAAAGATGC | 55**°C** |
| 86 | RM23332 | 8 | 23.80 | CAACCCTCCATCACTCACTTCTACC | GATGCATCCAGAGACCAAAGACC | 55**°C** |
| 87 | Hv09-07 | 9 | 4.35 | CATCTCAGCAAACAAGAACA | GTAAAGACTCCAGCTTTCTCC | 55**°C** |
| 88 | RM219 | 9 | 7.88 | CGTCGGATGATGTAAAGCCT | CATATCGGCATTCGCCTG | 55**°C** |
| 89 | RM24311 | 9 | 14.80 | CCTTTGGTTAGCTCTTGGATTTGC | GCATGCTTGCCATCACTTAGC | 55**°C** |
| 90 | RM3700 | 9 | 15.42 | AAATGCCCCATGCACAAC | TTGTCAGATTGTCACCAGGG | 55**°C** |
| 91 | RM288 | 9 | 18.56 | CCGGTCAGTTCAAGCTCTG | ACGTACGGACGTGACGAC | 55**°C** |
| 92 | RM201 | 9 | 20.17 | CTCGTTTATTACCTACAGTACC | CTACCTCCTTTCTAGACCGATA | 55**°C** |
| 93 | RM5095 | 10 | 0.05 | CTATATGACTATGCGAATGG | ACAAATGCAACTAAGGTAGA | 55**°C** |
| 94 | RM222 | 10 | 2.62 | CTTAAATGGGCCACATGCG | CAAAGCTTCCGGCCAAAAG | 55**°C** |
| 95 | RM25012 | 10 | 3.40 | TCTGACTCATCTTGACGGACAGG | GAGGGAGTCGAGGAAGGAGAGG | 55**°C** |
| 96 | RM25352 | 10 | 13.30 | GTTCCCAAATTTCCAGTAGTGACG | CCGAGGTCGATCAGTGAAAGG | 55**°C** |
| 97 | RM36 | 10 | 14.53 | CAACTATGCACCATTGTCGC | GTACTCCACAAGACCGTACC | 55**°C** |
| 98 | RM228 | 10 | 22.20 | CTGGCCATTAGTCCTTGG | GCTTGCGGCTCTGCTTAC | 55**°C** |
| 99 | RM286 | 11 | 0.38 | GGCTTCATCTTTGGCGAC | GGCTTCATCTTTGGCGAC | 55**°C** |
| 100 | RM1812 | 11 | 2.39 | CAGCTAGTGAGCTCCTAGTG | GCTAACCCACCAACTTATTC | 55**°C** |
| 101 | RM552 | 11 | 4.81 | CGCAGTTGTGGATTTCAGTG | CGCAGTTGTGGATTTCAGTG | 55**°C** |
| 102 | RM26281 | 11 | 6.41 | AACAAATCCCACATGACGATGC | GCCCTAGTCAGTCCCTCTGTAATCC | 55**°C** |
| 103 | RM202 | 11 | 9.00 | CAGATTGGAGATGAAGTCCTCC | CCAGCAAGCATGTCAATGTA | 55**°C** |
| 104 | RM7226 | 11 | 13.93 | GCGGCGTATTAGCGTTGTAC | CCATAAGGTTCTAGCCCATG | 55**°C** |
| 105 | RM26746 | 11 | 16.82 | GAAGGCCAGAACAATTCTATCACG | CCTAGGAGCGTTTGTAGGTGTGC | 55**°C** |
| 106 | RM20 | 12 | 0.90 | ATCTTGTCCCTGCAGGTCAT | GAAACAGAGGCACATTTCATTG | 55**°C** |
| 107 | RM19 | 12 | 1.09 | CAAAAACAGAGCAGATGAC | CTCAAGATGGACGCCAAGA | 55**°C** |
| 108 | RM247 | 12 | 3.10 | TAGTGCCGATCGATGTAACG | CATATGGTTTTGACAAAGCG | 55**°C** |
| 109 | RM2935 | 12 | 7.42 | CAGCAAATTTGTTACTTATG | TGCTATGTTTTTTTATAACG | 55**°C** |
| 110 | RM1246 | 12 | 19.26 | CTCGATCCCCTAGCTCTC | TCACCTCGTTCTCGATCC | 55**°C** |
| 111 | RM277 | 12 | 22.30 | CGGTCAAATCATCACCTGAC | CAAGGCTTGCAAGGGAAG | 55**°C** |
| 112 | RM28636 | 12 | 25.10 | CCATCAACTTCTCCAGCTTACTCG | TAGAAGCGACGTAATGCAGAACC | 55**°C** |
